# Essential Oil Nanoemulsions—A New Strategy to Extend the Shelf Life of Smoothies

**DOI:** 10.3390/foods13121854

**Published:** 2024-06-13

**Authors:** Alicja Napiórkowska, Amin Mousavi Khaneghah, Marcin Andrzej Kurek

**Affiliations:** 1 Department of Technique and Food Development, Warsaw, Institute of Human Nutrition Sciences, Warsaw University of Life Sciences, Nowoursynowska 159c, bud. 32, pok. 109B, 02-787 Warszawa, Poland; marcin_kurek@sggw.edu.pl; 2 Halal Research Center of IRI, Iran Food and Drug Administration, Ministry of Health and Medical Education, Tehran 1435713715, Iran; mousavi.amin@gmail.com

**Keywords:** smoothie, essential oils, nanoemulsions, food preservation, consumers acceptability

## Abstract

**Highlights:**

**What are the main findings?**

- The use of essential oils in food is still limited.
- Emulsification reduces impact on sensory properties of essential oils.
- Essential oils are characterized by numerous health-promoting, antimicrobial and antioxidant properties.

**What is the implication of the main finding?**

- Essential oil nanoemulsions as promising ingredient for extending the shelf life of smoothies.

**Abstract:**

Over the years, consumer awareness of proper, healthy eating has increased significantly, but the consumption of fruits and vegetables remains too low. Smoothie drinks offer a convenient way to supplement daily diets with servings of fruits and vegetables. These ready-to-eat beverages retain the nutritional benefits of the raw ingredients from which they are made. Furthermore, they cater to the growing demand for quick and nutritious meal options. To meet consumer expectations, current trends in the food market are shifting towards natural, high-quality products with minimal processing and extended shelf life. Food manufacturers are increasingly aiming to reduce or eliminate synthetic preservatives, replacing them with plant-based alternatives. Plant-based preservatives are particularly appealing to consumers, who often view them as natural and organic substitutes for conventional preservatives. Essential oils, known for their antibacterial and antifungal properties, are effective against the microorganisms and fungi present in fruit and vegetable smoothies. However, the strong taste and aroma of essential oils can be a significant drawback, as the concentrations needed for microbiological stability are often unpalatable to consumers. Encapsulation of essential oils in nanoemulsions offers a promising and effective solution to these challenges, allowing for their use in food production without compromising sensory qualities.

## 1. Introduction

Consumers are increasingly prioritizing the health and wellness impact of products when making purchasing decisions. They favor foods and beverages with high nutritional value, a natural appearance, and fresh flavors. Additionally, they seek products that not only offer nutritional benefits but also provide a convenient way to consume recommended servings of vegetables and fruits. As a result, smoothie drinks have seen a surge in popularity worldwide in recent years. In Germany, for instance, the daily consumption of smoothies increased from 650,000 people in 2018 to 820,000 in 2021, marking a 26% rise [1]. This trend is mirrored in Poland, where smoothie consumption escalated from 8 million liters annually in 2012 to 11 million liters in 2018, representing a 37.5% increase [2]. Smoothies are appealing due to their convenience and are suitable for consumption at home, at work, and while traveling. Furthermore, they serve as an excellent source of vitamins and minerals and are viewed as a preferable alternative to juices because they retain the fiber present in the raw ingredients. Compared to fruit and vegetable juices, smoothie consumption promotes satiety and positively impacts health [3,4,5]. Moreover, smoothies present a viable option for children, particularly those who are resistant to eating vegetables. Research conducted by Rollins et al. [6] demonstrates that preschool-aged children readily accept fruit and vegetable smoothies, effectively fulfilling 34–41% of their daily fruit and vegetable intake requirements with this beverage.

Smoothie drinks are semi-liquid products characterized by a soft consistency and distinctive viscosity. They are crafted from ripe and fresh fruits and/or vegetables, typically in the form of juices and purees, and may include fruit or vegetable particles [7]. Occasionally, other additives such as yogurt, milk, or honey are incorporated into the mixture during the blending process [8]. The production process begins with the creation of cloudy juice and puree, which are subsequently blended. Initially, the fruit and vegetable raw materials undergo washing, cutting, and crushing to achieve a uniform pulp. For cloudy juice production, the crushed raw material is pressed, and the resulting juice is packaged, deaerated (at temperatures of 55–60 °C and a pressure of 0.5 to 0.6 MPa), pasteurized, cooled, and stored. Conversely, in puree production, steaming follows the crushing of raw materials. During this step, the pulp is heated (fruits at 70–90 °C, vegetables at 90–100 °C) to deactivate enzymes that catalyze the oxidation or decomposition of pectin substances. Steaming also softens the raw material, facilitating subsequent grinding. The grinding process yields a finely comminuted puree and allows for the separation of woody parts such as seeds or pips. The puree is then homogenized, leading to aeration, which can influence the product’s sensory characteristics and create favorable conditions for microbial growth. Following homogenization, the puree undergoes pasteurization and storage. To prepare a smoothie drink, juice and puree are mixed, followed by homogenization, de-aeration, and pasteurization [8,9].

Thermal stabilization of smoothies can induce alterations in sensory attributes such as color, flavor, and palatability, as well as impact the functional properties of the product through the initiation of various chemical reactions. Consequently, thermal treatment may influence the content of vitamin C and other antioxidants. As a result, there has been an expansion in consumer demand for unpasteurized smoothies over the past decade. Consequently, an increasing number of smoothies available in the market are produced by pressing fruit and/or vegetable juice (involving washing, cutting, grinding, and pressing), creating fruit and/or vegetable puree (involving washing, cutting, and blending), and then amalgamating the resulting semi-finished products and fixing them through a non-thermal process such as HPP (High-Pressure Pasteurization) [8,9,10]. However, we are witnessing the phenomenon of food technology neophobia—consumers exhibit apprehension towards new food preservation technologies. The introduction of novel technologies is a necessary element of food engineering and production, offering numerous benefits in terms of nutrient preservation, quality, and food safety. The adoption of such technologies heavily relies on consumer acceptance. The findings of a study by Guzik et al. [11] indicated that conventional methods of food preservation (i.e., pasteurization) are better perceived and accepted by consumers due to their familiarity. Despite this, it was also affirmed that consumers hold a positive attitude towards HPP [11,12]. Nevertheless, it’s crucial to acknowledge that bacterial spores exhibit high resistance to this technology, as they can still germinate even after exposure to pressures of 1000 MPa. Thus, inactivation requires either combining high pressure (400–600 MPa) with a high temperature (90–120 °C) or applying pressure in multiple cycles [13,14,15]. Furthermore, extreme HPP conditions can lead to protein denaturation, alteration of physical properties, oxidation of polyunsaturated fatty acids, and acceleration of Maillard reactions [16].

Other non-thermal technologies that prolong the shelf life of food without necessitating high temperatures or pressures are available. One such approach involves utilizing natural bioactive compounds with antioxidant and/or antimicrobial properties as substitutes for chemical preservatives [11]. Essential oils, for instance, fulfill these criteria as they have demonstrated both antioxidant and antimicrobial properties, although they are not presently employed for this purpose [17,18].

**Figure 1:**
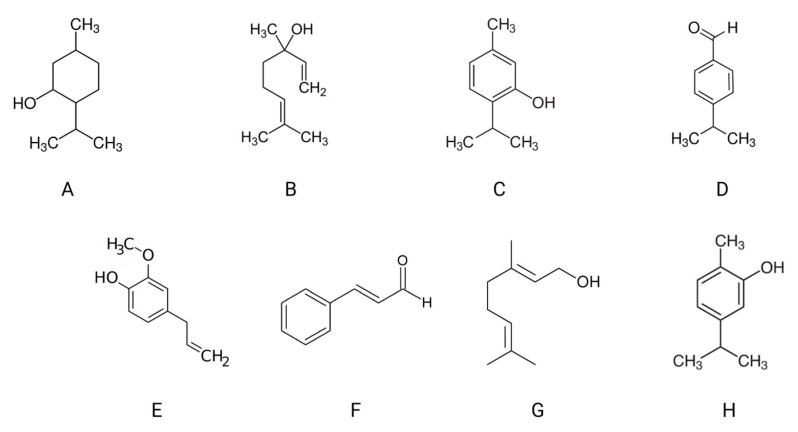
Examples of the main ingredients of essential oils: menthol (**A**), linalool (**B**), thymol (**C**), cuminaldehyde (**D**), eugenol (**E**), Cinnamaldehyde (**F**), myrcene (**G**), and carvacrol (**H**) (own elaboration).

Essential oils (EO) are secondary metabolites synthesized by oil-producing plants belonging to families such as *Pinaceae*, *Zingiberaceae*, and *Lauraceae*. They consist of multicomponent combinations comprising up to several hundred compounds, with the main components including terpenes, aldehydes, ketones, phenols, alcohols, and others (Figure 1) [18,19,20,21]. Furthermore, certain components of essential oils exhibit health-enhancing effects, such as the anticancer properties of cuminaldehyde, the anti-inflammatory effects of eugenol, the analgesic properties of menthol, and digestive benefits [22,23]. Thyme essential oil, extracted from *Thymus vulgaris* L., predominantly contains thymol (up to 45%), carvacrol (approximately 25–60%), borneol (about 8–15%), and linalool (about 8%), which impart disinfectant and expectorant properties to thyme extracts. Peppermint oil extracted from *Mentha piperita* L. serves not only as a bacteriostatic agent but also as a choleretic in various preparations. Moreover, it interacts with cold receptors on the skin and mucous membranes, inducing a cooling sensation. The primary constituent of peppermint oil, menthol (comprising over 50%), is responsible for this effect. Another renowned essential oil is lemon balm oil, derived from the leaves of *Melissa officinalis* L. This essential oil possesses a spasmolytic (antispasmodic) effect, calming the central nervous system, and exhibits bacteriostatic and virostatic properties. The predominant components of lemon balm oil are terpene compounds, namely citral and citronelal, which constitute approximately 40% of the oil [22,23].

Hence, a pertinent question arises regarding consumer acceptance of the incorporation of essential oils into foods. A study by Vital et al. [24] reveals that 70% of respondents are familiar with essential oils, with a notable 82.2% perceiving them as natural substances. Moreover, 57.8% of respondents expressed a willingness to pay a premium for products enriched with essential oils (EOs). Many essential oils are included in the U.S. Food and Drug Administration’s (FDA) GRAS (Generally Recognized as Safe) list for use in food. Approved oils encompass mint, rosemary, oregano, basil, lavender, sage, cinnamon, clove, laurel, lemon, orange, and ginger [25,26]. This suggests that products like smoothies containing essential oils could enjoy widespread consumer acceptance.

This article delves into the antimicrobial and antioxidant properties of essential oils, exploring their potential application in smoothie drinks, with a focus on their impact on the characteristic microflora of such beverages, along with associated limitations.

## 2. Essential Oils Are Effective against the Microflora Characteristic of Smoothies

Smoothies can be contaminated by various microorganisms present in fresh fruit and vegetable juices and purees. These contaminants, including bacteria, yeasts, and molds, originate from the natural microflora of the raw ingredients or are introduced during production. Table 1 outlines the types of microbiological contamination commonly found in minimally processed fruit and vegetable juices, reflecting the microbial risks associated with smoothies.

### 2.1. Bacteria

The predominant microflora in smoothie-type beverages typically includes Gram-negative rods, particularly *Pseudomonas* spp., *Enterobacter* spp., *Erwinia* spp., and *Xanthomonas* spp., along with lactic acid bacteria, yeasts (*Saccharomyces* spp.), and molds such as *Fusarium*, *Aspergillus*, *Penicillium*, or *Alternaria*, which may produce mycotoxins such as aflatoxins, ochratoxins, patulin, or aspergillic acid. Pathogenic bacteria that may be present in such products include *Aeromonas hydrophila*, *Escherichia coli* O157: H7 (in apple juices), *Salmonella* spp. (mostly in citrus juices), *Yersinia enterocolitica*, *Campylobacter jejuni*, *Shigella* spp., *Staphylococcus aureus*, *Bacillus cereus*, and *Listeria monocytogenes*, often introduced from equipment surfaces. Additionally, *Cryptosporidium* parasites, causing cryptosporidiosis (mainly in apple juices), may be found. Pasteurized smoothies may contain lactic acid bacteria (imparting an undesirable buttery taste, typically from *Leuconostoc* and *Lactobacillus*), as well as acetic acid-producing bacteria (*Acetobacter* and *Gluconobacter*), resulting in diacetyl production with a distinctive unpleasant taste. Spore-forming bacteria of the *Alicyclobacillus* genus and *Byssochlamys fulva* molds, capable of surviving thermal processing, may also be present [36,37].

In studies investigating the impact of various essential oils on *Leuconostoc citreum* in tomato juice, Lee et al. [38] demonstrated that oregano and thyme oils, at a concentration of 0.3125 µL/mL, completely inhibited the growth of these bacteria. Carvalho et al. [39] explored the effect of lemon balm oil (*Melissa officinalis* L.) on *Listeria monocytogenes* in watermelon juice, testing a concentration of 4xMIC (4 × 0.5 µL/mL), which led to complete inhibition of the bacterium from day 2 to day 7, contrasting with a 1 log10 (CFU/mL) increase observed in the growth control. Notably, not only herb-derived essential oils are effective against juice-spoiling bacteria; citrus oils such as lemon or tangerine EO have also demonstrated efficacy. de Souza Pedrosa et al. [40] revealed that at a sensory-accepted dose of 0.5 μL/mL, these oils exhibited a multifaceted effect in inactivating *L. brevis* and *L. mesenteroides* cells in orange juice. This effect involved DNA damage, disruption of membrane integrity, adverse impacts on membrane permeability and polarization, as well as suppression of metabolic and efflux activities. Furthermore, Lin et al. [41] investigated four different vegetable juices (cucumber, carrot, spinach, and bitter gourd) contaminated with *Escherichia coli* O157: H7. They found that concentrations of 3 mg/mL (MIC) and 6 mg/mL (MBC) reduced the population of these bacteria by 50.71% and 99.99%, respectively, compared to the control.

#### *Alicyclobacillus* spp.

Among the prevalent microorganisms in smoothies, the *Alicyclobacillus* genus warrants special attention. These Gram-positive, non-pathogenic, thermoacidophilic bacteria thrive in acidic environments (pH 1.5–6.5) and temperatures ranging from 20–70 °C. Being spore-formers, they can withstand pasteurization, posing a significant challenge in fruit and vegetable drink production. *Alicyclobacillus* is commonly found in juices and beverages derived from various fruits such as tomatoes, apples, pears, oranges, grapefruits, mangoes, blackcurrants, chokeberries, raspberries, and others [29,42,43,44]. The presence of *Alicyclobacillus* in the final product leads to the development of an unpleasant taste characterized as medicinal, disinfectant, or smoky, attributed to the production of guaiacol by these bacteria [37,45,46,47]. One prominent species, *Alicyclobacillus acidoterrestris*, exhibits robust spore survival, enduring pasteurization at 90 °C for 6–23 min, depending on juice type. Recent studies suggest that *A. acidoterrestris* spores can be inactivated at 115 °C within 8 min. However, employing such parameters in pasteurization adversely affects the organoleptic and nutritional qualities of the juice or smoothie. Detecting contamination with *Alicyclobacillus* spp. is challenging as they do not produce gas during growth, leading to no packaging swelling or significant pH changes in the product. Consequently, product spoilage may occur before the expiration date, often unrecognized by producers until consumer complaints arise. Hence, preventing the proliferation of *Alicyclobacillus* microorganisms is crucial, as their growth can result in substantial financial losses for producers [46,47].

Hence, it is imperative to explore novel methods for processing fruit and vegetable juices to ensure microbiologically safe products. Given the limitations of pasteurization in controlling *Alicyclobacillus* bacteria growth, research is underway on alternative inactivation methods such as Pulsed Electric Field (PEF), microwaves, ultrasounds, cold plasma, or high-pressure pasteurization [37]. Additionally, there is growing interest in utilizing essential oils (EO) as natural preservatives for *Alicyclobacillus*. Several research teams have investigated the effectiveness of various plant extracts (e.g., lemon, rosemary, grape seed) for this purpose [44,48,49,50,51,52,53].

Maldonado et al. [50] explored the impact of essential oils on *Alicyclobacillus acidoterrestris* spore germination, comparing it with conventional preservatives such as potassium sorbate and sodium benzoate. Sodium benzoate at doses of 0.1–0.5 g/L completely inhibited spore germination, while potassium sorbate at a dose of 0.6 g/L only delayed germination by two days. Higher concentrations (up to 1.2 g/L) extended the stagnation phase to 7 days. Lemon essential oil, in concentrations of 0.08%, 0.12%, and 0.16%, completely inhibited *A. acidoterrestris* spore germination throughout the 11-day study. However, Huertas et al. [49] reported that essential oils might confer a protective effect on bacterial spores, including those produced by *Alicyclobacillus*, thereby enhancing their resistance to temperature. *A. acidoterrestris* bacteria and spores were subjected to pasteurization at 80–100 °C (with a D95 °C of 2.26 min). Pre-pasteurization addition of citral and limonene significantly increased the D95 °C values to 3.17 min and 4.17 min, respectively. The authors concluded that these oily compounds could augment the heat resistance of microorganisms.

### 2.2. Yeasts

Fruit and vegetable juices, including smoothies, are commonly vulnerable to spoilage by yeast genera such as *Candida*, *Pichia*, *Rhodotorula,* and *Saccharomyces*. Despite unfavorable conditions such as low pH, high sugar concentration, and refrigeration, yeast can proliferate in these products, leading to significant organoleptic changes. The sweet, acidic nature (pH approximately 3.0–3.5) and relatively anaerobic environment make them prone to fermentation spoilage by yeast. The microbiological stability of smoothies primarily results from heat treatment (pasteurization) and the addition of preservatives such as benzoic acid. During yeast growth, undesirable byproducts such as carbon dioxide can cause packaging to swell, distort, or even explode. Yeast also produces alcohol and releases pectinases, leading to flocculation, phase separation, and increased turbidity of the product. In liquid products, yeast growth is evident as a biomass film on the surface or as sediment at the bottom of the package [22,35,54].

Leneveu-Jenvrin et al. [32] found that *Thymus leptobotrys* essential oil (TlEO) at a concentration of 0.25‰ completely inhibited the growth of *Rhodotorula mucilaginosa*, but this inhibition effect was alleviated at a concentration of 0.05‰, with yeast growth comparable to the control. Regarding *Thymus maroccanus* essential oil (TmEO), the growth of *R. mucilaginosa* was inhibited to a certain extent, with percentages of 43.7% and 37.5% of the control, respectively, at concentrations of 0.25‰ and 0.05‰. Additionally, *S. cerevisiae* growth was completely inhibited at TmEO 0.25‰ and partially inhibited (29.4%) in the presence of TlEO 0.25‰, as well as TlEO or TmEO at 0.05‰. These differences in effectiveness against the same yeasts were attributed to variations in chemical composition. While both essential oils contained similar amounts of carvacrol, thymol, and p-cymene, TmEO had notably higher levels of α-pinene, α-terpinene, and myrcene.

### 2.3. Molds

Filamentous fungi, commonly known as molds, pose a significant threat to smoothie producers, leading to substantial economic losses when they develop in the finished product. Despite rigorous production standards, food spoilage and fungal contamination persist as serious issues, particularly in developing countries. Similar to yeast, molds can thrive in low pH environments with high sugar concentrations, with some capable of producing heat-resistant spores. The spoilage of fruit and vegetable smoothies is often attributed to molds from genera such as *Aspergillus*, *Penicillium*, *Mucor*, *Alternaria*, *Cladosporium*, and *Botrytis*, as well as *Neosartorya* spp., *Talaromyces* sp., *Byssochlamys*, and *Eupenicillium* spp. [30,55,56]. These fungi commonly contaminate fruits such as apples, tomatoes, oranges, pineapple, strawberries, and mangoes, often introduced by insects, birds, soil, or water. While the pasteurization process effectively reduces the growth of heat-sensitive molds, the presence of molds post-pasteurization suggests contamination after processing or the use of subpar-quality fruits and vegetables. However, the challenge lies in heat-resistant molds such as *Neosartorya* spp., *Talaromyces* sp., *Byssochlamys*, and *Eupenicillium*, which survive pasteurization and cause spoilage characterized by unpleasant taste and odor, gas formation, drink clarification, or visible mycelium on the surface [30,57].

Even in small concentrations, essential oils have demonstrated the ability to inhibit mold growth in food. Guerra et al. [58] found that both 2.5 µL/mL and 1.25 µL/mL concentrations of peppermint oil from different mint species were equally effective in inhibiting mold growth, with no statistical differences observed in the results. The study showed inhibition of mold species such as *Botrytis cinerea*, *Penicillium expansum*, *Rhizopus stolonifer*, and *Aspergillus niger* by 90.2% to 97.5%. However, for inhibiting spore germination, the concentration of 2.5 µL/mL was more effective (86.1% to 90.4%) compared to 1.25 µL/mL (77.2% to 79.5%). Mustard and clove essential oils, in vapor form, have also been shown to inhibit *Botrytis cinerea* growth on strawberries [59]. In vitro results demonstrated a 100% inhibition of the mycelial growth of *Colletotrichum gloeosporioides* when treated with savory and thyme essential oils. In in vivo experiments on papaya fruits, the application of savory and thyme oils at a concentration of 2000 µL L^−1^ resulted in a reduction in lesion diameter by 59.26% and 58.40%, respectively, and a decrease in fruit decay by 64.07% and 54.82%, respectively [60].

In pineapple juice, *Penicillium citrinum* and temperature-resistant *Talaromyces amestolkiae* were completely inhibited by 0.25‰ thymol (the main component of thyme essential oil). Even a five-fold reduction in concentration resulted in their growth to only 16% and 17%, respectively, compared to the control sample [32]. Thus, essential oils can effectively reduce mold growth in juices and smoothies. The critical issue, however, is their effect on inhibiting mold development during storage. According to industry standards, a juice is considered fit for consumption when the total mold and yeast count does not exceed 6 log CFU/mL. In the case of pineapple juice, the addition of pure thymol (0.25‰, 0.05‰) and essential oils from two species of thyme (0.25‰, 0.05‰) extended the juice’s expiry date by 14 days (with CFU/mL after 14 days lower than 6 log) [32]. Table 2 illustrates the effect of some essential oils on extending the microbiological stability of juices.

Indeed, the presence of fungi in food poses more than just organoleptic deterioration of the product. Consumer safety may also be compromised by mycotoxins, which are produced by certain types of mold [30,31,61].

**Table 2:** Examples of using essential oils as an antimicrobial and antioxidant agent in juices with sensory evaluation.

Juice	Essential Oil	Obtained Results	References
Orange	sesame (*Sesamum indicum*)	Proved antibacterial activity against *Staphylococcus aureus*, prolonged shelf life of orange juice	[62]
	sweet orange (*Citrus sinensis*)	Reduce the treatment time to cause the inactivation of up to 5 Log_10_ cycles of *E. coli* O157:H7 Sakai cells. The sensory analysis carried out showed that flavor, color, and odor were accepted by the panelists—the parameters were rated above 6 on the hedonic scale.	[63]
	*Thymbra capitata* L.	Growth inhibition of *E. coli* up to 20 days, *L. monocytogenes,* and *S. aureus* up to 15 days of storage at 5 °C	[64]
	cinnamon (*Cinnamomum verum* J.Presl)	Reduction of *E. coli* O157:H7 and *Salmonella enteritidis* by more than 6 log	[65]
Apple	lemongrass (*Cymbopogon citratus*), mandarin (*Citrus reticulata*)	Reduction the *E. coli* population by more than 5 log-reduction after being in contact for 28 days	[66]
	sweet orange (*Citrus sinensis*)	Reduction the treatment time to cause the inactivation of up to 5 Log_10_ cycles of *E. coli* O157:H7 Sakai cells	[53]
	cinnamon (*Cinnamomum verum* J.Presl)	Reduction of *E. coli* O157:H7 and *Salmonella enteritidis* by more than 6 log	[65]
Strawberry	*Solidago canadensis* L.	Effective inhibition of the growth of *Botrytis cinerea*	[67]
	cinnamon (*Cinnamomum verum* J.Presl)	Reduction of *E. coli* O157:H7 and *Salmonella enteritidis* by more than 6 log	[65]
Pineapple	mint (*Mentha piperita* L.)	The reduction of *C. albicans* by more than 3 log CFU/mL, *C. tropicalis* by almost 2 log CFU/mL, *P. anomala* by almost 4 log, and *S. cerevisiae* by 4 log CFU/mL in 72 h. The attributes of appearance, odor, and viscosity were not affected (*p* > 0.05) by essential oil. Juice received hedonic scores varying between “like slightly” and “really enjoyed”.	[68]
	lemongrass (*Cymbopogon citratus* D.C. Stapf.)	Reductions in counts of *E. coli* and *L. monocytogenes* ≥5 log cycles after 1 h and 45 min, respectively; for *Salmonella enteritidis* the same reduction was confirmed after 12 h	[69]
Pear	cinnamon (*Cinnamomum verum* J.Presl)	Reduction of *E. coli* O157:H7 and *Salmonella enteritidis* by more than 6 log	[65]
Guava	mint (*Mentha piperita* L.)	Reduction of *C. albicans* by almost 3 log CFU/mL, *C. tropicalis* by 1 log CFU/mL, *P. anomala,* and *S. cerevisiae* by more than 6 log CFU/mL in 72 h. The attributes appearance, odor, and viscosity were not affected (*p* > 0.05) by essential oil. Juice received hedonic scores varying between “like slightly” and “really enjoyed”.	[68]
Lemon	lemon (*Citrus limon* L.)	Total inhibition of *Alicyclobacillus acidoterrestris* germination and outgrowth of spores under refrigerated storage over 11 d.	[50]
Mango	mint (*Mentha piperita* L.)	Reduction of *C. albicans* and *C. tropicalis* by almost 2 log CFU/mL, *P. anomala* by almost 4 log and *S. cerevisiae* by more than 2 log CFU/mL in 72 h. The attributes of appearance, odor, and viscosity were not affected (*p* > 0.05) by essential oil. Juice received hedonic scores varying between “like slightly” and “really enjoyed”.	[68]
Carrot	*Thymbra capitata* L.	*E. coli* O157:H7 count decreased after treatment	[70]

#### Mycotoxins Can Come along with Molds

Mycotoxins are secondary metabolites produced by filamentous fungi under specific temperature and humidity conditions. These substances maintain chemical stability even during processing at high temperatures (80–120 °C) and storage; thus, they may persist in products even after the fungi are removed. Because mycotoxins can cause serious intoxication at low doses, their presence in beverages is a significant health concern for consumers [35,71,72]. Fungal infections in fruits and vegetables occur through contact with soil, air, and water used for irrigation, posing a risk of mycotoxin contamination. Environmental stressors such as drought, mechanical damage, pests, and unfavorable weather conditions further increase this risk. After harvesting, mycotoxin-producing fungi can contaminate raw materials and finished products during storage, packaging, transportation, processing, and washing [71,73]. To mitigate mycotoxin presence in food, three main strategies have been adopted: preventing raw material contamination before and after harvest, inactivating mycotoxin in food, and inhibiting mycotoxin absorption in the gastrointestinal tract [71].

Patulin (PAT) is one of the most common and toxic mycotoxins found in food, produced by various species of fungi, predominantly *Penicillium* spp. molds. Among these, *Penicillium expansum* and *P. griseofulvum*, often found in juices, are significant producers of patulin. *Penicillium expansum* molds can produce patulin within the temperature range of 0–24 °C with a minimum water activity of 0.99. Additionally, fungi from the genera *Aspergillus* and *Byssochlamys* are also capable of producing patulin. The presence of patulin in fruits and vegetables poses challenges for the food industry as it is thermostable and highly soluble in low pH environments such as fruit juices. Moreover, PAT-producing fungi are commonly found in visually appealing fruits and vegetables, particularly those based on apples, pears, grapes, and oranges. These fungi have also been isolated from smoothies containing various fruits and vegetables, such as tomatoes, carrots, beets, mangoes, strawberries, and pineapples, among others [27,71,72,74].

The use of antifungal agents to inhibit mold growth is a crucial strategy for enhancing food safety and reducing microbial mycotoxin production. Natural antifungal substances, including essential oils, are emerging as promising candidates in this regard. A study by Prakash et al. [75] revealed that essential oils might be more effective in reducing mold growth, thereby minimizing the risk of mycotoxin production, compared to commonly used chemicals. They compared the efficacy of myrrh, coriander, and marjoram essential oils with that of ascorbic acid and gallic acid against the toxigenic strain of *Aspergillus flavus*. Minimum inhibitory concentration (MIC) and minimum fungicidal concentration (MFC) were determined, showing that the essential oils were more effective in inhibiting the development of *A. flavus* (MIC = 3 μL/mL for myrrh and marjoram EO, MIC = 2.5 μL/mL for coriander EO) than ascorbic and gallic acids (MIC > 10 μL/mL). Several studies have investigated the impact of essential oils on the growth of *Fusarium* spp., *Aspergillus* spp., and *Penicillium* spp., as well as their mycotoxin production [75,76,77,78]. These studies suggest that essential oils may reduce mycotoxin production, and their inhibitory activity is dose-dependent [77,78]. Notably, hop essential oil achieved nearly complete inhibition (98.3–99.9%) of deoxynivalenol (DON) produced by *F. graminearum* isolates [79]. Similarly, cinnamon oil significantly inhibited patulin production by *P. expansum*, reducing its accumulation to less than one-third compared to the control group [80]. These findings demonstrate the potential of essential oils to reduce mycotoxin production by molds.

## 3. Essential Oils—Effective against Enzymatic Oxidation of Smoothies

Enzymatic browning in smoothies is a significant concern due to the mechanical stress and cellular delocalization of polyphenol oxidase (PPO) and substrates during processing [81]. This accelerates the oxidation of phenolic substances, leading to the formation of brown, black, or red pigments known as melanin [81,82,83]. The enzymatic browning not only affects the visual appeal of smoothies but also alters their taste and nutritional quality, forming undesirable compounds such as furfural and 5-hydroxymethylfurfural [81,84,85,86]. Additionally, oxidative reactions catalyzed by lipoxygenase (LOX) further compromise the nutritional quality of smoothies, leading to the degradation of essential fatty acids, vitamins, and proteins, as well as off-flavor formation and color loss due to carotene and chlorophyll degradation [76,84]. To address these challenges, protecting smoothies from oxidative spoilage and controlling enzymatic browning are essential for extending their shelf life. Various studies emphasize the importance of managing enzymatic browning to maintain the quality of smoothies and meet consumer preferences [76,87].

The shift towards plant-based antioxidants in food systems is driven by concerns over the carcinogenicity of synthetic antioxidants such as butylated hydroxytoluene (BHT), butylated hydroxyanisole (BHA), and tertiary butylhydroquinone (TBHQ). Plant antioxidants offer not only benefits for food products but also for human health and the environment [76,88]. Among oxygen radicals, the hydroxyl radical is particularly reactive, making it crucial to target in antioxidant interventions. Studies suggest that essential oils, such as those from *Moringa oleifera*, exhibit higher scavenging effects against oxygen radicals compared to commonly used synthetic antioxidants such as trolox, ascorbic acid, BHT, and BHA [89]. This highlights the potential of plant-based antioxidants, such as essential oils, to provide effective oxidative protection for both food products and human health.

The effectiveness of essential oils in preserving the sensory quality of fruit and vegetable juices, commonly used as bases for smoothies, has been demonstrated in several studies. A study by Khan et al. [90] showed that longan fruit fumigated with thymol was more resistant to enzymatic browning than the control sample. After 6 days of storage, the browning index for the control sample was over 4.5, while for the sample treated with thymol, it was about 2.5. Similarly, the 0.05% addition of essential oils such as lemongrass, clove, rosemary, basil, and sage effectively reduced color changes in apple juice during storage [91]. Pineapple juice supplemented with 0.25‰ or 0.05‰ of thymol, *Thymus leptobotrys* essential oil, or *Thymus maroccanus* essential oil showed no significant alterations in color parameters after storage [32]. Additionally, sensory evaluations of vegetable juices (cucumber, carrot, spinach, and bitter gourd) treated with *Litsea cubeba* essential oil revealed that while storage time influenced sensory perception, differences between treated and untreated samples were not significant after one day at room temperature [41]. These findings underscore the potential of essential oils to extend the sensory quality and shelf life of fruit and vegetable juices, thereby enhancing their suitability as smoothie ingredients. Table 2 shows the effect of some essential oils on extending the sensory quality of juices.

### 3.1. Limitations on the Use of Essential Oils in Smoothies

The practical application of essential oils in food products is hindered by several factors, including their chemical composition, which can vary widely depending on factors such as geographical origin, plant species, agricultural practices, and extraction methods [92,93,94]. For example, studies have identified different chemotypes of rosemary with varying compositions of key compounds such as 1,8-cineole or camphor [95]. Moreover, agricultural techniques, seasonal variations, and extraction methods further contribute to the variability in essential oil composition [93,94,95]. Additionally, there is a limited understanding of how essential oils interact with other food ingredients [61], which complicates their application in food matrices. Despite their demonstrated efficacy as antibacterial, antifungal, and antioxidant agents [20,24,96,97,98,99], addressing these challenges is essential for the wider adoption of essential oils in food preservation and enhancement.

Taban et al. [94] investigated the impact of different extraction methods on the chemical composition of laurel (*Laurus nobilis* L.) essential oil extracted from its leaves. Using techniques such as hydrodistillation, steam-water distillation, microwave-assisted hydrodistillation, and ohm-assisted hydrodistillation, they analyzed the EO’s composition via gas chromatography coupled with mass spectrometry (GC/MS). Their findings revealed variations in the content of key compounds depending on the extraction method employed. For instance, the eucalyptol content ranged from 5.07% to 34.37%, while the α-terpinenyl acetate, erpinene-4-ol, and sabinene contents also varied. Interestingly, ohm-assisted hydrodistillation yielded the highest concentrations of eucalyptol, erpinene-4-ol, and linalool at 50.07%, 6.02%, and 2.72%, respectively, compared to other methods.

The efficacy of essential oils ©n stabilizing food products microbiologically often diminishes upon their transfer to in situ conditions. This necessitates higher EO concentrations to achieve microbial stabilization. Interactions between the hydrophobic components of EOs and fats, starches, or proteins in food matrices can affect their antimicrobial activity. Notably, smoothies lack a fat phase, eliminating this interaction. The water activity (a_w_) of the product also plays a crucial role; lower aw values hinder EO penetration into bacterial cells. Moreover, the availability of nutrients in food may expedite bacterial cell repair. The antimicrobial activity of EOs is influenced by pH, with lower pH values enhancing their hydrophobicity and facilitating diffusion into bacterial cell membranes. Temperature and initial microbial contamination levels further impact EO activity. In food matrices, EO concentrations often need to be doubled to achieve efficacy comparable to in vitro studies, but this can result in sensory issues due to the strong taste and smell of EOs [20,24,100,101,102].

### 3.2. Essential Oil Nanoemulsions—Solution for Those Limitations

The utilization of essential oils in the form of nanoemulsions presents a promising solution to the challenges encountered in food production, including their incorporation into smoothies. Nanoemulsions, characterized by particle diameters ranging from 10 to 100 nm, offer unique physicochemical properties conducive to food applications [102,103]. Their small droplet size renders nanoemulsions translucent and light-transmitting, ensuring minimal impact on the color of smoothies [104,105]. Additionally, nanoemulsions exhibit enhanced resistance to gravitational separation and particle aggregation compared to traditional emulsions [103,105,106]. Oil-in-water (o/w) nanoemulsions have emerged as effective carriers of antimicrobial compounds in food systems. The reduced droplet size facilitates the migration and attachment of nanoemulsion droplets to bacterial cell walls, leading to the destabilization of lipid fractions within the cell membranes [102]. This mechanism enhances the antimicrobial efficacy of essential oils encapsulated in nanoemulsions, thereby addressing the challenges associated with their practical application in food production.

**Figure 2:**
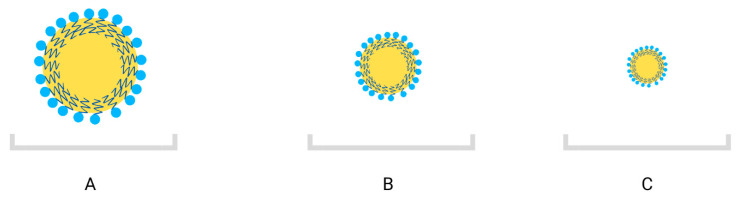
Droplet sizes of macroemulsion: >500 nm (**A**), microemulsion: <500 nm (**B**), and nanoemulsion: 10–100 nm (**C**) (based on [113], own elaboration).

Indeed, nanoemulsions of essential oils offer several advantages that make them highly suitable for food applications. One key benefit is their enhanced physical stability within the food matrix, which protects the essential oils from interactions with other ingredients and maintains their efficacy as preservatives. Additionally, nanoemulsions help mask the strong taste and odor of essential oils, improving their sensory acceptability among consumers [102,103,107]. Moreover, the reduced droplet size of nanoemulsions facilitates their even distribution throughout the product, enhancing their efficiency as antimicrobial agents (Figure 2) [100,108,109]. Nanoemulsions, with their increased active surface area due to smaller droplet diameter, exhibit superior antimicrobial properties compared to conventional emulsions with larger droplets [110,111,112]. This improved antimicrobial efficacy further contributes to the preservation of food products and extends their shelf life.

## 4. Comparison of Essential Oils and Essential Oil Nanoemulsions for Extending Smoothie Shelf Life

To measure the shelf life of smoothies enhanced with essential oil nanoemulsions, a combination of microbiological, sensory, and physicochemical analyses is required. These analyses are performed over time to determine the effectiveness of the nanoemulsions in extending the product’s shelf life.

Microbiological analysis involves monitoring the growth of spoilage microorganisms and potential pathogens using standard methods, such as plate count techniques, on selective media. Regular sampling over a set period, typically weekly, allows for the assessment of microbial stability. In a typical experimental setup, smoothies containing essential oil nanoemulsions are stored at refrigeration temperatures (4–6 °C) and monitored over a period of 4 to 8 weeks. Sensory analysis includes evaluating changes in taste, odor, and appearance, conducted by trained panelists to ensure consumer acceptability. Physicochemical properties such as pH, viscosity, and color are also measured periodically to track any changes that might affect product quality [114,115,116]. 

The research conducted so far consistently demonstrates that essential oil nanoemulsions exhibit greater efficacy in controlling the development of various microorganisms compared to their bulk counterparts (Table 3). For instance, cinnamon essential oil in nanoemulsion form induced larger zones of inhibition against bacteria such as *Bacillus subtilis* and *Staphylococcus aureus* compared to bulk EO, and nanoemulsions also exhibited significantly lower minimum inhibitory concentrations (MIC) [117]. In a study examining the interaction of pure or emulsified *Thymus daenensis* EO with *Escherichia coli* over 60 min, it was found that nanoemulsions were more effective in reducing bacterial viability. While pure EO reduced the bacterial cell count to around 8 log CFU/mL within an hour, nanoemulsions of the same EO eliminated viable cells after just 5 min [118]. Interestingly, EO nanoemulsions may demonstrate varying effectiveness depending on the composition of the EO, as shown in research by Wan et al. [78]. Their study tested the impact of nanoemulsions of cinnamon, clove, mint, lemongrass, and thyme essential oils on the growth of *Fusarium graminearum*. Significant differences were observed in the effects of individual EO nanoemulsions, with thyme oil nanoemulsion being the most effective, having an EC90 value of 7.61 mg/g, while mint oil nanoemulsion was the least effective, with an EC90 value of 23.67 mg/g. Nanoemulsions of essential oils are effective not only against bacteria but also against fungi. Research conducted by Liu et al. [119] demonstrated that lemongrass essential oil nanoemulsion was more effective than pure oil in inhibiting the growth of *Aspergillus flavus*. However, the antimicrobial properties of essential oil nanoemulsions depend on their composition, including the content of essential oil, water, emulsifier, type, and size of the emulsion droplets [120]. There are documented cases in the literature where EO nanoemulsions, despite having antimicrobial effects, were less effective than pure EO. For example, research conducted by Özogul et al. [121] showed that laurel essential oil was more effective against *Klebsiella pneumoniae* (MIC = 12.5%) and *Staphylococcus aureus* (MIC = 12.5%) than its nanoemulsion (MIC = 25% and MIC > 25%, respectively).

The above leads to the conclusion that, in practical terms, the shelf life of smoothies can be extended by several days to weeks depending on the type and concentration of essential oil used, as well as storage conditions. However, more research is needed to better understand the specific parameters that these nanoemulsions should possess to maximize their effectiveness. Future studies should focus on optimizing the formulation process, including the selection of emulsifiers, the ratio of essential oil to water, and the ideal droplet size. Additionally, investigations into scalable production methods and the stability of these nanoemulsions under various storage conditions are essential. Such research will provide valuable insights into the practical applications of essential oil nanoemulsions in food preservation and ensure their efficacy and safety in commercial products. 

## 5. Future Perspectives, Challenges, and Conclusions

Smoothies are gaining popularity these days. At the same time, we are facing an increase in consumer interest in low-processed products without chemical additives. The food industry was therefore faced with the challenge of searching for new methods of preserving food products, including smoothies.

A promising method of preserving smoothies seems to be the addition of essential oils, which are characterized by a high rate of inactivation of microorganisms and antioxidant properties. Despite having these features, there are limitations to their wide-scale application. Finding an amount of essential oil nanoemulsion additive that guarantees microbiological stability and is sensory acceptable at the same time is now the biggest challenge. A promising solution in this matter seems to be in the form of nanoemulsion. 

At present, there is a significant lack of research results concerning the activity of essential oils in food matrices. Most of the research is based on the effects of essential oils directly analyzed against microorganisms isolated from food. To our knowledge, there have been no studies on smoothie drinks. It is necessary to conduct a number of studies to determine whether the use of essential oil nanoemulsions for smoothie preservation would be possible. These studies should focus not only on issues related to the safety of beverages preserved in this way but also on the degree of acceptability of finished products by consumers—taste, aroma, and color. Currently, there has not been much research conducted on the sensory analysis of products with the addition of essential oils in pure form or in the form of nanoemulsions. Knowing the answers to these issues seems to be the key goal of introducing essential oils into food preservation on a larger scale.

## Figures and Tables

**Table 1:** Various types of microbiological contamination in minimally processed fruit and vegetable juices.

Juice	Bacteria	Mold	Yeast	References
Apple	*Alicyclobacillus acidoterrestris*, *Escherichia coli* O157: H7, *Bacillus* spp., *Streptomyces* spp., *Staphylococcus* spp.	*Penicillium expansum*, *Penicillium griseofulvum*, *Talaromyces* spp., *Byssochlamys* spp.	*Blastomyces* sp., *Rhodotorula rubra*, *Saccharomyces cerevisiae*, *Candida stellata*, *Candida glabrata*, *Pichia anomala*	[27,28,29,30]
Orange	*Alicyclobacillus acidoterrestris*, *Bacillus cereus*, *Bacillus megaterium*, *Pseudomonas aeruginosa*, *Escherichia coli*	*Aspergillus* sp., *Trichoderma* sp., *Penicillium islandicum*, *Geotrichum* spp.	*Saccharomyces cerevisiae*, *Rhodotorula mucilaginosa*, *Candida mesenteric*, *Blastomyces* sp., *Candida stellata*, *Candida glabrata*, *Pichia anomala*	[29,30,31]
Pineapple	*Alicyclobacillus acidoterrestris*, *Escherichia coli*, *Listeria monocytogenes*, *Salmonella enteritidis*	*Penicillium expansum*, *Penicillium griseofulvum*, *Penicillium citrinum*, *Talaromyces amestolkiae*, *Byssochlamys* spp.	*Saccharomyces cerevisiae*, *Rhodotorula mucilaginosa*, *Candida stellata*, *Candida glabrata*, *Pichia anomala*	[27,29,30,32]
Tomato	*Bacillus* spp., *Enterobacter* spp., *Klebsiella* spp., *Proteus mirabilis*, *Escherichia coli*, *Alicyclobacillus acidoterrestris*	*Aspergillus niger*, *Penicillium expansum*, *Penicillium griseofulvum*	*Saccharomyces cerevisiae*, *Pichia* spp., *Candida* spp., *Hanseniaspora valbyensis*	[27,29,33,34,35]

**Table 3:** Comparison of the antimicrobial properties of pure essential oils and their nanoemulsions.

Microorganism	Essential Oil	Inhibition Zones [mm]	MIC [%]	Essential Oil Nanoemulsion	Inhibition Zones [mm]	MIC [%]	Reference
*Bacillus subtilis*	cinnamon (*Cinnamomum verum* J.Presl)	12.0 ± 0.6	0.130	cinnamon (*Cinnamomum verum* J.Presl)	18.0 ± 1.3	0.080	[117]
*Proteus vulgaris*		13.0 ± 0.8	0.130		20.0 ± 1.5	0.085	
*Klebsiella pneumoniae*		11.0 ± 0.7	0.400		18.0 ± 1.4	0.250	
	laurel (*Laurus nobilis* L.)	17.25 ± 0.65	12.50	laurel (*Laurus nobilis* L.)	7.75 ± 0.65	25.00	[121]
*Staphylococcus aureus*	cinnamon (*Cinnamomum verum* J.Presl)	10.0 ± 0.4	0.130	cinnamon (*Cinnamomum verum* J.Presl)	17.0 ± 1.2	0.075	[117]
	lemon myrtle (*Backhousia citriodora* F. Muell)	-	0.156	lemon myrtle (*Backhousia citriodora* F. Muell)	-	0.062	[122]
	laurel (*Laurus nobilis* L.)	10.75 ± 0.25	12.50	laurel (*Laurus nobilis* L.)	15.00 ± 0.91	>25.00	[121]
*Pseudomonas aeruginosa*	cinnamon (*Cinnamomum verum* J.Presl)	9.0 ± 0.3	0.500	cinnamon (*Cinnamomum verum* J.Presl)	16.0 ± 1.0	0.300	[117]
	lemon myrtle (*Backhousia citriodora* F. Muell)	-	0.156	lemon myrtle (*Backhousia citriodora* F. Muell)	-	0.031	[122]
*Escherichia coli*	lemon myrtle (*Backhousia citriodora* F. Muell)	-	0.625	lemon myrtle (*Backhousia citriodora* F. Muell)	-	0.25	

## Data Availability

No new data were created or analyzed in this study. Data sharing is not applicable to this article.
